# Advancing whole-of-government approaches to tobacco control: Article 5.3 and the challenge of policy coordination in Bangladesh, Ethiopia, India and Uganda

**DOI:** 10.1136/tobaccocontrol-2021-057154

**Published:** 2022-02-11

**Authors:** Rachel Ann Barry, S M Abdullah, Aastha Chugh, Selamawit Hirpa, Praveen Kumar, Denis Male, Rob Ralston, Tracey Wagner-Rizvi, Jeff Collin

**Affiliations:** 1 Global Health Policy Unit, University of Edinburgh, Edinburgh, UK; 2 Department of Economics, University of Dhaka, Dhaka, Bangladesh; 3 Research and Development, Ark Foundation, Dhaka, Bangladesh; 4 HRIDAY, New Delhi, India; 5 Department of Preventive Medicine, School of Public Health, Addis Ababa University, Addis Ababa, Ethiopia; 6 Department of Commerce, Manipal Academy of Higher Education, Manipal, Karnataka, India; 7 Department of Food Technology and Nutrition, Makerere University, Kampala, Uganda; 8 SPECTRUM (Shaping Public Health Policies to Reduce Inequalities and Harm), Edinburgh, UK

**Keywords:** global health, low/middle-income country, public policy, tobacco industry

## Abstract

**Introduction:**

Despite an extensive evidence base on the diverse economic, environmental and social benefits of tobacco control, difficulties in establishing coordinated national approaches remain a defining challenge for Framework Convention on Tobacco Control (FCTC) implementation. Minimising tobacco industry interference is seen as key to effective coordination, and this paper analyses implementation of Article 5.3 guidelines, exploring implications for whole-of-government approaches to tobacco control in Bangladesh, Ethiopia, India and Uganda.

**Methods:**

Based on 131 semistructured interviews with government officials and other key stakeholders, we explore barriers and facilitators for promoting: (1) *horizontal coordination* across health and other policy spheres, and (2) *vertical coordination* across national and subnational governments on Article 5.3 implementation.

**Results:**

Our analysis identifies common barriers to coordination across diverse geographical contexts and varying approaches to implementation. They highlight broadly shared experiences of limited understanding and engagement beyond health agencies; restricted responsibility and uncertainty amid conflicting mandates; tensions with wider governance practices and norms; limited capacity and authority of coordination mechanisms; and obstacles to vertical coordination across local, state and national governments. Interview data also indicate important opportunities to advance coordination across sectors and government levels, with Article 5.3 measures capable of informing changes in practices, building support in other sectors, allowing for ‘bottom-up’ innovation and being shaped by engagement with civil society.

**Conclusion:**

Supporting effective implementation of Article 5.3 is key to advancing multisectoral approaches to FCTC implementation and tobacco control’s contributions to global health and sustainable development.

## Introduction

Stimulating new multisectoral engagement across government departments was a key feature of negotiations for the WHO Framework Convention on Tobacco Control (FCTC),^1^ and effective collaboration across sectors is essential to its comprehensive implementation.^2^ Yet, despite an extensive evidence base regarding the diverse economic, environmental and social benefits of tobacco control,^3–5^ difficulties in establishing coordinated national approaches remain a defining challenge amid competing interests and mandates.^2 6 7^

This paper uses frameworks drawn from public administration and policy studies^8–10^ to analyse barriers and opportunities for promoting policy coordination in tobacco control, with a focus on Article 5.3 implementation in Bangladesh, Ethiopia, India and Uganda. The term policy coordination here refers to efforts that ensure activities and agendas across different parts of government do not undermine each other, promoting what Peters defines as governance whereby ‘decisions in one program or organization consider those made in others and attempt to avoid conflict’.^8^ Promoting the active engagement of all relevant ministries in advancing tobacco control requires a focus on ‘joined up’^10^ or ‘whole-of-government’ approaches.^11^ A whole-of-government approach entails ‘government agencies working across portfolio boundaries to achieve a shared goal and an integrated government policy, programme or service response to particular issues’.^12^ Such coordination issues operate across *horizontal* and *vertical* dimensions. Horizontal coordination describes the promotion of synergies across sectors, encompassing different ministries, agencies and policies within the same level of government (usually national). The vertical dimension denotes challenges of promoting coordination in interactions across diverse levels of government.^13^ Such challenges are often magnified in political systems with significant levels of policymaking autonomy at subnational levels characterised by multilevel governance.^10 14 15^ The FCTC seeks to support states in advancing horizontal coordination across sectors via general obligations requiring Parties to develop ‘comprehensive multisectoral national tobacco control strategies, plans and programmes’ (Article 5.1a) and ‘a national coordinating mechanism or focal points for tobacco control’ (Article 5.2). While the FCTC text addresses coordination across national, regional and international levels, attention to the vertical dimension within countries is restricted to its foreword acknowledging the need for commitment to the FCTC to ‘spread to national and local levels’.^16^

A focus on implementation of Article 5.3 and its guidelines offers a promising lens through which to explore challenges of coordination in tobacco control governance. This reflects both the priority afforded to minimising tobacco industry interference for rapid acceleration of FCTC implementation,^17^ and its significance for developing effective national coordination strategies.^2^ Article 5.3 guidelines seek to promote whole-of-government implementation across both sectors (specifying applicability ‘in all branches of government that may have an interest in, or the capacity to, affect public health policies with respect to tobacco control’) and levels (encompassing ‘national, state, provincial, municipal, local or other public or semi/quasi-public’ institutions or agencies).^18^ Yet, Article 5.3 also poses significant challenges to coordination and engaging other ministries, reflecting its embodiment of a very distinctive model of governance within public health.^19^ An emphasis on limiting government–industry interactions challenges longstanding practices in most government departments. It also runs counter to broader governance practices of stakeholder engagement and of collaboration and partnership with commercial actors.^20–22^

This paper aims to analyse efforts to implement Article 5.3 and associated guidelines, exploring implications for coordination in tobacco control within Bangladesh, Ethiopia, India and Uganda. It identifies common barriers to coordination across diverse geographical contexts and varying approaches to implementing Article 5.3. This highlights broadly shared experiences of limited understanding and engagement beyond health agencies; restricted responsibility and uncertainty amid conflicting mandates; tensions with wider governance practices and norms; limited capacity and authority of coordination mechanisms; and obstacles to vertical coordination across local, state and national governments. It then identifies opportunities to accelerate progress across both minimising industry interference and enhancing coordination, before discussing implications for advancing international FCTC implementation in the context of progressing broader commitments to the United Nations Sustainable Development Goals (SDGs).

## Methods

### Case selection

This research was undertaken within the Tobacco Control Capacity Programme, an international consortium funded by the UK Global Challenges Research Fund (GCRF). The wider project sought to develop new studies focused on tobacco control through a process of co-creation of research topics. It was through this process that Article 5.3 was identified by local research teams and via stakeholder events as a key research and policy priority across four countries—Bangladesh, Ethiopia, India and Uganda. Alongside a core focus on national implementation, research conducted by two project teams in India (Delhi and Manipal, Karnataka) allowed for examination of dynamics across multilevel governance (with the Delhi team exploring national and state-level efforts and the Manipal team focused on districts).

This paper builds upon linked case studies examining context-specific challenges of tobacco control governance in Bangladesh,^23^ Ethiopia,^24^ Uganda,^25^ the Indian state of Karnataka,^26^ and the dynamics of implementation across India’s states and union territories.^27^ Our case selection covers diverse tobacco control experiences and trajectories via which to understand coordination challenges and opportunities associated with Article 5.3 implementation. National coordination mechanisms (NCMs) have been established in all four countries, with India also allowing for coordination at subnational levels via state tobacco control cells and district-level coordination committees. Coverage of Article 5.3 implementation guidelines varies notably across these diverse contexts. In Uganda^28^ and Ethiopia,^29^ legislation broadly addresses most of the eight key recommendations of Article 5.3, while notably omitting reference to raising awareness of industry interference and treatment of state-owned elements of the tobacco industry. Ethiopia’s legislation also excludes reference to avoiding preferential treatment for the industry.^24 25^

While both policies are applicable across government departments, India’s national Code of Conduct for Public Officials is limited to the Ministry of Health & Family Welfare.^30^ This code provides for raising awareness but its measures omit preferential treatment, state-owned interests or regulation of corporate social responsibility (CSR) initiatives. This absence of CSR mirrors the majority of subnational notifications that have been adopted, including Karnataka’s state policy^27^ (though CSR is addressed by two districts: Udupi and Bengaluru (Rural)).^26^ Conversely, in Bangladesh, measures seeking to implement Article 5.3 measures have not been adopted. While the National Tobacco Control Cell has developed a draft code of conduct and guidelines, the process of approving such measures has stalled.^23^ Civil society monitoring reports indicate varying levels of tobacco industry interference across the four countries; of 57 countries in a 2020 ranking report, for example, Uganda and Ethiopia were identified as high performing states (ranked 3rd and 10th overall), while rankings for India and Bangladesh were less encouraging (31st and 41st).^31^

### Data collection and analysis

This study draws on 131 semistructured interviews with key stakeholders engaged in tobacco control, including officials from health and other relevant ministries (ie, trade, agriculture, finance, revenue and customs, environmental protection and law enforcement), legislators, non-governmental organisations and health advocates. Our sample includes a larger number of health officials and advocates than other policy actors, reflecting varying degrees of participation in tobacco control and challenges of participant recruitment in the COVID-19 pandemic, particularly in India and Bangladesh. While overall the primary focus was on national-level policy implementation, state-level and district-level initiatives in India allowed for examination of dynamics across multiple levels of government, as reflected in the distribution of interviewees. Tables 1 and 2 provide a summary of the distribution of interviewees, separated by country and by location and role.

**Table 1:** Interviewees by country and by level of government

Location	Bangladesh	Ethiopia	India	Uganda	Total
National	14	20	4	34	70
State (eg, regional)	–	1	30	–	31
Local (eg, districts)	1	–	26	1	28
Total	15	21	60	35	**131**

**Table 2:** Interviewees by country and role

Sector	Bangladesh	Ethiopia	India	Uganda
Health	1	4	24	4
Non-health agencies	3	11	20	20
*Trade*	–	1	–	3
*Agriculture*	–	–	3	2
*Finance & Revenue*	3	1	5	2
*Customs*	–	3	–	–
*Development*	–	–	–	2
*Food & Drug Administration*	–	6	–	–
*Education*	–	–	5	2
*Environment*	–	–	–	4
*Other government agencies*	–	–	7	5
Executive office	–	1	1	–
Elected officials	–	1	–	1
NGOs and think tanks	7	1	10	8
Academic researcher	3	1	3	1
International organisation	1	2	2	1
Total	15	21	60	35

NGOs, non-governmental organisations.

The interviews were semistructured and conducted using an interview guide organised around three core themes: awareness of Article 5.3 and its norms; perceptions about government–industry interactions; and how rules and procedures had been operationalised across government departments and levels (including a focus on coordination). The semistructured approach allowed for both thematic consistency and scope for adaptation across political and institutional contexts, with interviewees also asked context-specific questions.

While data collection in Ethiopia and Uganda (July–September 2019) was completed before the COVID-19 pandemic, fieldwork in India (Delhi: January 2019–October 2020, and Manipal: July 2020–April 2021) and Bangladesh (February–July 2020; April–May 2021) was significantly impacted by the pandemic and mitigation measures. Travel restrictions and officials having additional policy responsibilities limited availability of potential respondents, and researchers switched to conducting interviews via telephone or video conferencing.

Country-based researchers carried out and transcribed the interviews. All interviews in Uganda and most in India were conducted in English, with others in Amharic (21), Bengali (15) and Kannada (a regional language in Karnataka, 11). Interviews ranged from 15 to 95 min, with average duration between 25 and 40 min. Interviews were coordinated by SH, AC, DM, PK and AS, and transcripts coded by SH, AC, DM, PK, AS, RR, TW-R and RAB with oversight from JC. Transcripts were coded in NVivo V.12 using an interpretative approach, with themes and subthemes developed iteratively and refined through repeated readings of interview text. Coded data were then used to develop a thematic analysis organised around barriers and facilitators of coordination on Article 5.3 across horizontal and vertical levels.

Emergent findings were discussed at GCRF consortium meetings in Delhi, India and Addis Ababa, Ethiopia in early 2020, and developed thereafter via conference calls.

## Results

While the cases were selected on the basis of significant differences in implementation of Article 5.3 measures, the interview data demonstrated notable consistency in identifying six broad barriers to effective coordinated action.

### Limited awareness and understanding beyond health officials

Interview data from across all four countries indicated that familiarity with Article 5.3 among government officials was largely restricted to those working in ministries of health and related health agencies, a finding consistent with previously published work.^21 32^ Furthermore, detailed knowledge of its requirements and implications was largely concentrated among those directly engaged in tobacco control and centred in the NCMs. While some sensitisation activities were reported in all contexts, there was widespread recognition that promoting whole-of-government engagement would require more extensive dissemination. Hence, one Ethiopian government official noted that more effective implementation would ‘need strong awareness-creation work’, while in the Indian state of Bihar, one health official highlighted the ‘need to conduct more sensitisation workshops’ to assist colleagues in integrating 5.3 measures ‘in their work practice’. An agriculture official in Uganda reported having expected greater outreach work from the Ministry of Health (MoH) following tobacco control legislation:

If they are interested in making the law more understandable, you go out to those actors whom you think are very instrumental in implementing the law and sensitise them about it, so they understand what it is, then in a way they become part of it.

Alongside the restricted involvement of other ministries, the interview data suggest that coordination may be impeded by limited understanding and engagement among some health actors. One Bangladesh interviewee envisaged progress as entailing that ‘a representative from the tobacco industry needs to be there because both sides must be there to implement this Article 5.3’. In India, some state and district officials regarded tobacco industry CSR activities as unproblematic: ‘If they sponsor [us] through CSR, let’s take it. What’s wrong with that?’ In Ethiopia, one legal advisor suggested such activities could promote health goals ‘by advancing against tobacco consumption’.

### Restricted responsibility amid divergent mandates

The lack of engagement with Article 5.3 measures by ministries beyond health was frequently explained via a view that responsibility for tobacco control was restricted to the MoH. Interviewees from health departments felt that such measures ought to be seen as entailing wider commitments across government and not, in the words of one India official, as ‘only the responsibility of the Health [Ministry]’. Similarly, an interviewee in Bangladesh noted of colleagues in other departments:

I have a very strong observation that they initially assume that this tobacco control issue is not their involvement; it is a matter for the Ministry of Health. But it is a matter of the whole state and it needs a comprehensive effort to control it. They don’t understand this thing.

Some non-health officials who recognised tobacco control issues as impacting on their responsibilities nevertheless did not consider Article 5.3 commitments as applicable in their roles. Hence, an official in Uganda’s labour ministry differentiated between relevant and irrelevant FCTC measures:

The aspects of my work related to tobacco control are on another Article, not Article 5.3 basically. The [FCTC] basically has many other provisions including one that looks at alternatives to tobacco in agriculture. So, for us as a country, I am responsible for the control of crop pests and diseases.

Differing perspectives on Article 5.3 were also reflected in varying attitudes regarding appropriate engagement with the tobacco industry. Minimising interactions was presented as incompatible with the industry’s legal status by a Karnataka tax official: ‘[e]verybody has the liberty to interact with us, [to] take our suggestion with respect to the law and its implications’. Similarly, in Uganda, an agriculture official defended engagement with the tobacco industry and advancing its interests:

[Our role] is to support the private sector; to do their business. So, if a private company comes and approaches my department…I have no problem because tobacco growing is not prohibited in our country… So, if somebody comes up and says that he wants to develop that industry, it is my responsibility as a department to support industry growth.

### Lack of capacity in coordination mechanisms

Concerns about the capacity of intersectoral coordination committees to effectively engage other government departments in implementing Article 5.3 were raised by interviewees across all four countries. Coordination mechanisms are based in ministries of health and in practice, cross-sectoral engagement tends to be infrequent and for specific purposes, as in Bangladesh where ‘interactions happened only when there is a need’. State-level empowered committees throughout India were described as having been set up to meet only following complaints about industry interference, rather than establishing regular information exchange to promote more institutionalised engagement. Interviewees often linked limited capacity directly to constraints on financial and human resources. In Uganda, one health official noted that Article 5.3 implementation ‘needs resources but the resources are not there’, and a health advocate in Bangladesh stated: ‘our National Tobacco Control Committee (NTCC) is very weak. They do not have the capacity to work at the policy-making stage.’ One Ethiopia official presented NCM resource constraints as inhibiting a whole-of-government approach to minimising industry interference:

A strong institutional system is needed for tobacco control; it can’t be one department. We need strong institutional mechanisms. Maybe it is difficult to have an independent institute due to financial reasons but we need an independently operating system within a certain institute.

### Tensions with wider governance practices and norms

Challenges to Article 5.3 implementation were further compounded by perceived tensions with commitments to stakeholder consultation in policy development. This was most explicitly evident in Ethiopia, with one health official describing how broader procedural requirements meant that the tobacco industry had to be consulted when developing legislation that would impact on it:

[T]hat means they are one of the stakeholders… So, we need to hear what they say concerning the law and we need to explain them how they are going to implement it and if they have any reasonable points, we need to entertain that.

A Uganda trade official similarly suggested that governance practices entailed tobacco control being ‘a collaborative effort’, since for ‘any policy to be passed through the Cabinet Secretariat, it has to have had… sector-wide consultation with all relevant parties so that they agree on what is being developed.’

Alongside conflicts with wider governance norms and practices, prospects for coordinated implementation of Article 5.3 measures can also be affected by specific policies. In India, practices around managing industry interference are circumscribed by a requirement in its Companies Act that large businesses allocate 2% of net profits to CSR actions.^33^ Interviewees reported difficulties in reconciling this legislative requirement with Article 5.3 implementation guidelines that specify denormalising and regulating tobacco company CSR activities.

### Vertical challenges: coordination across national, state and local government

Interviewees emphasised heightened challenges of navigating complex institutional environments, particularly in India, where the federal political system allocates responsibilities for public health across national, state and district levels.^34^ Complex multilevel governance can pose barriers to accessing and disseminating information and practices on limiting government–industry interactions. According to one civil society representative:

I think it would be very, very difficult, because it’s not just one person deciding; there is a whole system. There are so many layers, you don’t know who is doing what. It may be at district-level or you know sub-district level that something is happening.

Similar themes were evident in Ethiopia, reflecting devolved authority to subnational levels. One central government official saw challenges confronting the NCM in promoting coordinated implementation as highlighting the need for ‘technical and financial support’ to extend to subnational government:

Because regions are the one which has a big role on enforcement of the law. For example, if we talk about smokefree, regions are the one that give licences to restaurants so they can have a big role in controlling enforcement of the law. So, we need to empower them by giving training, developing the infrastructure and human power should be strengthening at regional level.

### Catalysts for improved coordination

In all four countries, the interview data primarily highlighted the breadth and significance of barriers to developing whole-of-government approaches to Article 5.3 implementation. Yet none of these settings were presented as so hostile or difficult as to make the prospect of such coordination unattainable. Instead, potentially significant opportunities to shape policy and practice were identified by diverse interviewees across Bangladesh, Ethiopia, India and Uganda.

### Positive impacts on practice

In all four countries, there were strong indications that Article 5.3 measures were partially redefining government–industry interactions. Interviewees emphasised limits to such changes, and saw them as confined to health officials, but the policy environment was nonetheless seen as changing. In India, state health officials across Mizoram, Bihar and Himachal Pradesh highlighted how measures adopted by these states had empowered them to restrict interactions, while providing for improved transparency. In Bangladesh, where NTCC attempts to adopt Article 5.3 measures have stalled, interviewees described how increased sensitisation meant that health officials no longer engaged with the tobacco industry. This was contrasted with both high levels of engagement in other ministries and previous MoH practices. One health advocate recalled how, in establishing a committee to develop the 2005 tobacco control legislation, ministry officials had ‘invited the tobacco company to the committee. They wanted [British American Tobacco (BAT) Bangladesh] to cooperate with the government in enacting the law’.

### Enthusiasm for Article 5.3 norms and practices beyond health

Alongside acknowledgement of limited awareness and understanding of Article 5.3 measures beyond health, there were encouraging signs of enthusiasm in diverse departments where officials did engage. In Karnataka, district officials in administration, education and police departments voiced opposition to any government involvement in tobacco industry CSR activities. In Uganda, an official in the Ministry of Gender, Labour and Social Development embraced Article 5.3 measures as legitimating personal unease about engaging with tobacco companies, while a trade official noted that measures to promote transparency and avoid giving preferential treatment ‘will definitely link to the work we do on trade’.

### Strengthening coordination mechanisms

While interviewees were aware of the limitations of and constraints upon existing coordination mechanisms at national and subnational levels, these were seen as providing an important basis for progress. In Uganda, any criticisms of the Tobacco Control Committee were tempered by acknowledgement of its novelty, having been established in 2019, an event described by one health official as ‘a milestone on governance’. Elsewhere, there was recognition of scope for additional resources to generate progress. In Karnataka, a health official contended that the State Tobacco Control Cell could expand awareness across government departments with investment in a database and ‘a very good technical team’, while a Bangladesh health advocate asserted that more senior leadership for the NTCC such as ‘an Additional Secretary or Joint Secretary level coordinator’ would significantly enhance its impact.

### Bottom-up innovation

While acknowledging the coordination challenges of regulating government–industry interactions in India’s multilevel system, this context provided opportunities for subnational innovation, stimulating the wider adoption of policies. In Karnataka, for example, district-level notifications were seen as having paved the way for the state to act. One health official noted that ‘sometimes public health works through a bottom-up approach…some policy issues we cannot deal with directly with state-level senior officers for many technical reasons’, while a researcher suggested that in ‘[f]ollowing some role models or examples within their district, the state feels a bit more confident in enacting such provisions [as it indicates] higher buy-in.’

### Active engagement with civil society

The data also highlighted the role of civil society organisations (CSOs) in driving progress towards multisectoral participation in Article 5.3 implementation. This reflects recognition of their agenda-setting role in highlighting the need to tackle industry interference, including initiating legal action in Karnataka.^35^ One politician noted the impacts of such action:

The public interest litigation that happened to stop one of the meetings with the tobacco industry, which the government was supposed to participate in, was facilitated by [a health think tank]… Based on that, the state government formed its policy, then it spread to district administration.

Bihar’s policy was similarly described as ‘the outcome of strong collaboration between CSOs and government’, while in Bangladesh, a government official suggested that ‘without civil society nothing will happen’.

## Discussion

This analysis of the experiences of key informants across Bangladesh, Ethiopia, India and Uganda in seeking to implement Article 5.3 is broadly consistent with the challenges of multisectoral coordination identified in the limited empirical literature.^6 21 32 36^ It highlights the consistency with which engagement with Article 5.3 by government officials beyond health is restricted by limited awareness and understanding, as well as by perceived tensions with wider governance norms and with the mandates of other ministries. An interesting feature of the data is that few interviewees identified gaps in guideline recommendations adopted as inhibiting coordinated approaches to minimising industry interference. A small number of interviewees did comment on individual omissions, notably with respect to CSR, and the frequent citing of the need for more work in raising awareness arguably constitutes an implicit critique of existing policies and practices. However, interviewees did not reflect on the selective coverage of WHO implementation guidelines in policies adopted.

Incomplete adoption of Article 5.3 guidelines has previously been suggested to inhibit effective implementation, with one study identifying only 6% of Parties as having acted on over half of the recommendations,^32^ while subsequent implementation reports indicate limited further progress.^37^ Given the central problem of lack of engagement and familiarity beyond health agencies, the absence of commitments to raising awareness in policies adopted in the four countries appears as a serious omission. Similarly, developing a code of conduct for public servants applicable only to officials working within a health ministry^30^ seems likely to entrench rather than tackle the perceived limits in Article 5.3’s scope. Yet, evidence of gaps in awareness and understanding among health officials presented here highlights how the need for further sensitisation and broader engagement should not be assumed to apply only beyond ministries of health.

This study’s analysis of the vertical dimension of policy coordination illustrates the significance of challenges across local, state and national governments. Alongside the absence of such issues from the treaty text, these have received limited attention in studies of FCTC implementation and coordination.^17 36 38 39^ The data suggest that NCMs should pay greater attention to ensuring alignment with subnational tobacco control plans and governance practices.^2^ Nevertheless, evidence of policy learning across government levels in India highlights opportunities for policy innovation and diffusion in tobacco control that federal and devolved political systems can present, including in circumventing industry opposition.^40 41^ The recognition of opportunities for ‘bottom-up’ innovation sits alongside other potential catalysts for improved coordination via implementation of Article 5.3. In all contexts, the interview data do primarily emphasise barriers, but given the breadth of such challenges and Parties’ limited progress in adopting guidelines,^32^ the identification of positive ways forward and absence of pessimism about the feasibility of effective implementation are notable. These highlight a need for additional resources, including to support awareness raising and to increase the capacity of coordination mechanisms.

Increased recognition of the importance of strengthening governance to advancing FCTC implementation by Parties^37^ and funders^42^ is encouraging in this context. Yet, there remains a tendency for Article 5.3 implementation to be considered in isolation from wider governance issues addressed under Article 5. The interaction between dual challenges of minimising industry interference and of coordination explored here suggests that these require an integrated approach. This entails working to bridge gaps such as Article 5.3 guidelines not addressing the role of coordinating mechanisms in promoting cohesive approaches to conflict of interest management.

Though the polarised politics of tobacco control and dynamics of Article 5.3 do provide distinctive dimensions, the challenges of promoting policy coordination across sectors, stakeholders and levels of government are broadly shared with other issues in public policy and global health. Such problems are multiplied in the expansive policy agenda of the SDGs, where identifying scope for mutually reinforcing gains across policy spheres constitutes their defining challenge. In its ability to generate gains across health, economic, sustainable financing, environmental and gender objectives, tobacco control is well placed to advance the commitment (SDG 17.14) to ensure policy coherence for sustainable development. International agencies have recognised that ‘multisectoral coordination, legislation and protection against tobacco industry interference in policymaking are foundational to effective tobacco control’ and necessary to unlock tobacco control’s potential to accelerate sustainable development. This study highlights the governance challenges and opportunities that need to be addressed across Article 5.2 and 5.3 in order to effectively advance both FCTC implementation and the SDG agenda.

**What this paper adds**

**What is already known on this subject**

- Promoting effective multisectoral coordination constitutes a significant challenge confronting Framework Convention on Tobacco Control implementation and tobacco control governance.
- Article 5.3 implementation is central to advancing tobacco control, but its distinctiveness raises coordination issues in being viewed as inconsistent with wider governance practices of stakeholder engagement and industry consultation.

**What this paper adds**

- Despite substantive policy differences across the four low/middle-income country contexts, efforts to promote multisectoral approaches to protect health policy from industry interference face comparable challenges.
- Horizontal coordination on Article 5.3 with non-health ministries is hindered by limited understanding and engagement; restricted responsibility amid competing mandates; tensions with wider governance practices and norms; and the limited capacity and authority of coordination mechanisms.
- While differing responsibilities and policies across local, state and national governments create challenges for vertical coordination, efforts to implement Article 5.3 benefit from bottom-up innovation across government levels.
- Alongside broad barriers, interview data highlight opportunities to promote coordinated approaches to implementing Article 5.3, with potentially significant implications across health and sustainable development.

## Data Availability

No data are available.
